# Association of the use of e-cigarettes, combustible cigarettes or dual use with hypertension and mortality in hypertensive individuals: Insights from NHANES 2015–2018

**DOI:** 10.18332/tid/195397

**Published:** 2024-11-19

**Authors:** Yi Lu, Hao Jiang, Yin Ren, Meixiang Wang, Aili Yuan, Jing Wu, Zhongbao Ruan, Xiangwei Ding

**Affiliations:** 1 Department of Cardiology, The Affiliated Taizhou People’s Hospital of Nanjing Medical University, Taizhou School of Clinical Medicine, Nanjing Medical University, Taizhou, China

**Keywords:** e-cigarette, combustible cigarette, hypertension, NHANES, dual use

## Abstract

**INTRODUCTION:**

Combustible cigarettes have been shown to increase hypertension risk. Nevertheless, data on the association between electronic cigarettes (e-cigarettes), as well as dual use of e-cigarettes and combustible cigarettes, and hypertension, are limited.

**METHODS:**

This study aims to examine the association of the use of e-cigarettes, combustible cigarettes or dual use with hypertension. Data from the 2015–2018 National Health and Nutrition Examination Survey were used. Weighted logistic regression models were employed to determine the relationship between cigarette use and hypertension. Weighted Cox proportional hazard regression models were developed to evaluate the association between electronic/combustible cigarettes or dual use and mortality in hypertensive individuals.

**RESULTS:**

A total of 7696 participants (median age 47 years; 51.76% females) were included. In the adjusted model, the groups of e-cigarette use, combustible cigarette use, and dual use were found to be significantly associated with the risk of hypertension with AOR and 95% CI of 1.56 (1.01–2.42), 1.29 (1.01–1.64) and 1.83 (1.03–3.27) respectively. Significant trends of the relationship between cigarette use and hypertension were observed. The median follow-up for mortality was 38 months. Current e-cigarette use showed a positive correlation with all-cause death and cardiovascular death compared to never e-cigarette use with HR and 95% CI of 1.30 (1.01–1.66) and 1.30 (1.01–1.67), respectively. The trend of association of e-cigarette use with mortality was significant.

**CONCLUSIONS:**

This study shows that electronic/combustible cigarette use or dual use increased risk of hypertension. E-cigarettes were associated with a higher risk of all-cause mortality and cardiovascular mortality. Notably, the increased risk of mortality among e-cigarette users may be due to underlying, pre-existing comorbidities related to prior combustible cigarette use. Findings from the study provide evidence of the benefits of e-cigarette use control, especially among individuals with hypertension.

## INTRODUCTION

Hypertension is a significant global public health concern, imposing a substantial burden on individuals and societal well-being. According to the World Health Organization (WHO), more than 1.28 billion adults worldwide are affected by hypertension, with a disproportionate number residing in economically disadvantaged regions. In 2019, approximately 30% of the global population was diagnosed with hypertension, and the prevalence of this condition continues to increase^1^. It is commonly recognized that hypertension is a risk factor for a variety of health conditions, such as cerebral hemorrhage, diabetes mellitus, dyslipidemia, heart failure, and impaired kidney function^2^. In addition to increasing patients’ health risks, hypertension contributes to the rising cost of medical care, leading to limited economic activity and ultimately impeding economic prosperity. To minimize the impact of hypertension, while pharmacological treatments are recommended, prioritizing lifestyle changes, enhancing living conditions, and other non-pharmacological strategies may offer greater benefits in preventing disease progression^3^.

Previous studies have demonstrated that combustible cigarettes, containing nicotine as the primary chemical constituent, have been shown to elevate the likelihood of hypertension through multiple mechanisms such as endothelial dysfunction, systemic inflammation, and disrupted coagulation systems^4^. In addition to hypertension, a major risk factor for cardiovascular (CV) diseases, combustible cigarettes may substantially heighten the risk of CV morbidity and mortality^5^.

In response to the need for reducing combustible cigarette consumption, electronic cigarettes (e-cigarettes) were introduced as nicotine delivery devices with the intention of assisting in smoking cessation. Since their inception in 2003, there has been a discernible decrease in the prevalence of traditional cigarette use in the United States. Nevertheless, this technological advancement has substantially transformed the landscape of tobacco consumption^6^.

Following their introduction to the American market in 2007, the utilization of e-cigarettes has experienced a noteworthy increase, both domestically and internationally. In 2020, a study reported that >10 million individuals in the United States, representing more than 30% of smokers, were using e-cigarettes^7^. The safety of e-cigarettes as an alternative to traditional combustible cigarettes warrants additional research and examination. A study indicated that consistent use of e-cigarettes may assist individuals with hypertension in reducing or ceasing cigarette smoking, resulting in enhancements in systolic and diastolic blood pressure, as well as improved overall blood pressure management^8^. However, a cross-over study demonstrated that acute inhalation of e-cigarettes, both with and without nicotine, resulted in increased blood pressure^9^. Therefore, it is important to investigate the relationship between e-cigarette use and hypertension, as well as the potential long-term CV implications.

The objective of this nationwide study was to investigate the relationship between the use of electronic/combustible cigarettes or dual use and hypertension. Furthermore, this study aimed to elucidate the association of electronic/combustible cigarettes with all-cause mortality and CV mortality in individuals with hypertension.

## METHODS

### Study design and population

The National Health and Nutrition Examination Survey (NHANES) is a nationally population-based survey to assess the nutritional and health status of US children and adults. NHANES utilizes a complex multistage probability sampling design, collecting data from in-home interviews, physical measurements, and laboratory examinations with two-year cycles. The NHANES protocols were authorized by the ethics board of National Centre for Health Statistics (NCHC), and written informed consent was obtained from all adult individuals. The secondary analyses of NHANES data follow the NCHC guideline^10^.

**Figure 1:**
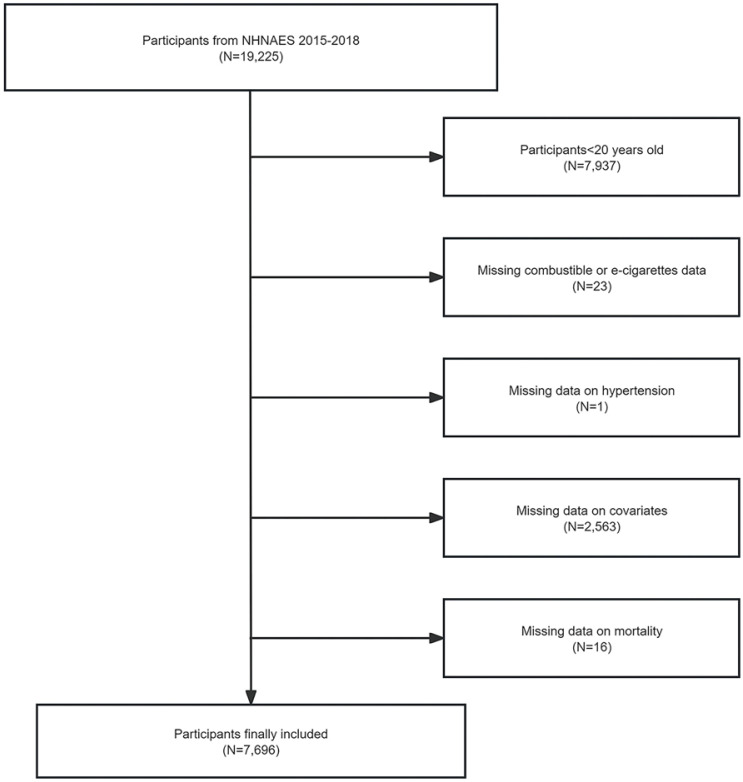
Flowchart of participants selection, NHANES 2015–2018, United States (N=7696)

The initial sample of this study was 19225 individuals, enrolled in two consecutive cycles from NHNAES 2015–2018. Participants aged <20 years (n=7937) were first excluded. Then participants without information on combustible or e-cigarettes (n=23), without information on hypertension (n=1), with missing covariates data (n=2563), and without information on mortality (n=16) were excluded. A total of 7696 individuals were included in the study (Figure 1).

### Assessment of electronic/combustible cigarette use or dual use

Combustible cigarette use was categorized into three groups, including never use, former use, and current use based on two questions: 1) ‘Have you smoked at least 100 cigarettes in your entire life?’ (SMQ020)^11^, and 2) ‘Do you now smoke cigarettes?’ (SMQ935). Never combustible use was identified as having smoked less than 100 cigarettes in the whole life. Former combustible cigarette use was identified as having smoked at least 100 cigarettes in the whole life, and not currently smoking cigarettes. Current combustible cigarette use was identified as having smoked at least 100 cigarettes in the whole life, and currently smoking cigarettes.

E-cigarette use status was categorized into three groups, including never use, former use, and now use on the basis of participants’ responses to these two questions: 1) ‘Have you ever used an e-cigarette or other electronic "vaping" product (these are battery-powered devices that usually contain liquid nicotine, and do not produce smoke), even just one time, in your entire life?’ (SMQ900); and 2) ‘During the past 30 days, on how many days did you use e-cigarettes?’ (SMQ905). Participants were considered never users if they responded ‘No’ to the prior questions. Former users were included if participants responded positively to the first question, and did not report even one time e-cigarette use in the past 30 days. Current e-cigarettes were participants who used e-cigarettes in their lifetime and used e-cigarettes in the past 30 days.

Dual use of e-cigarettes and combustible cigarettes was determined by e-cigarette use and combustible use status. Dual users were individuals who were both current e-cigarette users and current combustible cigarette users. E-cigarette-only user were participants who were current e-cigarette users, and never or former combustible cigarette users. E-cigarette-only users were categorized into those who were never combustible cigarette users, and those who were former combustible cigarette users. Combustible-only users were participants who were current combustible cigarette users, and never or former e-cigarette users. Never users were participants who were never combustible users and never e-cigarette users.

### Assessment of hypertension

Hypertension was determined based on in-home interviews and blood pressure (BP) measured in the mobile examination center (MEC). Participants were asked: ‘Have you ever been told by a doctor or other health professional that you had hypertension, also called high blood pressure?’ (BPQ202), and ‘Because of high blood pressure/hypertension, have you ever been told to take prescribed medicine?’ (BPQ040a). Trained health technicians performed BP measures using a calibrated Omron IntelliSense Blood Pressure Monitor. After resting in a sitting status for more than five minutes, three systolic BP and diastolic BP were measured, and the mean parameter of three BP was calculated. Participants were considered to have hypertension if they met any of the criteria according to the guideline: 1) Average systolic BP ≥140 mmHg; 2) Average diastolic BP ≥90 mmHg; 3) Self-reported hypertension; and 4) Ever prescribed antihypertensive medications^12^.

### Ascertainment of mortality

Mortality data of participants was ascertained by linkage to National Death Index (NDI) records, which matched death certificates (https://www.cdc.gov/nchs/data-linkage/mortality-public.htm). The study included all-cause death and CV death according to the International Classification of Diseases, tenth version (ICD-10). All-cause mortality was all specific causes of death, and CV death was defined as death related to CV diseases, including rheumatic heart diseases (ICD-10 codes I00-I09), hypertensive heart disease (I11), hypertensive heart and kidney disease (I13), and ischemic and other heart diseases (I20-I51) according to the instruction of Centers for Disease Control and Prevention, NCHS (https://www.cdc.gov/nchs/data/dvs/2a_2016.pdf).

### Assessment of covariates

Sociodemographic factors, physical examination, and health-related factors that may confound the correlation between electronic/combustible cigarettes and hypertension were considered covariates. Covariates in the study included age, sex (male, female), race (non-Hispanic Black, non-Hispanic White, other Hispanic, Mexican American, or other race), education level (less than high school, high school diploma or higher than high school), marital status (married, divorced or living alone), family poverty-to-income ratio (PIR) (<1, 1–2, 3–4 or >4), body mass index (BMI), alcohol use (never, former, mild, moderate or heavy), diabetes mellitus (yes/no) and coronary heart diseases (yes/no).

Alcohol use was categorized into five groups according to the previous study^13^. Never alcohol users were individuals who had <12 drinks in their lifetime. Former alcohol users were individuals who had ≥12 drinks in 1 year and did not drink alcohol last year, or did not drink last year but drank ≥12 drinks in their lifetime. Mild alcohol users were individuals who had ≤1 drink per day for females or had ≤2 drinks per day for males, in the past year. Moderate alcohol users were individuals who had 2–3 drinks per day for females or had 3–4 drinks per day for males, in the past year. Heavy alcohol users were individuals who had >3 drinks per day for females or had >4 drinks per day for males in the past year Diabetes mellitus was defined as physician-diagnosed diabetes, hemoglobin A1 (HbA1c) ≥6.5%, fasting blood glucose ≥7.0 mmol/L, 2-h oral glucose oral tolerance test (OGTT) blood glucose ≥11.1 mmol/L, or prescribed diabetes medication or insulin^14^. BMI was calculated as kg/m^2^.

### Statistical analysis

Given the complex, multistage nature of NHANES, statistical analyses were performed based on NHANES weighting recommendations. We conducted weighted analyses following 2-year examination weight (WTMEC2YR), primary sampling units (SDMVPSU), and strata (SDMVSTRA). In the study, continuous variables with normal distribution are presented using mean ± standard deviation (SD). Conversely, those with non-normal distribution are presented using median and interquartile range (IQR). Categorical variables are described using numbers and proportions. Kruskal–Wallis H tests were used to test differences for continuous parameters between three types of e-cigarettes. Pearson’s chi-squared test was performed to calculate differences among categorical variables.

The multivariate models were built based on biological plausibility and previous studies^15,16^. Weighted logistic regression models were established to identify the association between electronic/combustible cigarette use or dual use and hypertension. Three models were constructed: Model 1 was adjusted for age, sex, and race; Model 2 was adjusted as for Model 1 plus marital status, education level, and PIR. Model 3 was adjusted as for Model 2 plus BMI, alcohol use, coronary heart disease, and diabetes mellitus.

Schoenfeld residual plots and the Schoenfeld test were utilized to assess proportion hazards assumption. Weighted Cox proportional hazard regression models were developed to evaluate the relationship between use of electronic cigarettes, combustible cigarettes, or dual use and mortality, including all-cause death and CV death. The models adjusted for all the covariates, including age, sex, race, marital status, education level, PIR, BMI, alcohol use, coronary heart disease, and diabetes mellitus. Logistic regression models are reported as adjusted odds ratios (AORs) and 95% confidence interval (CI). Cox proportional hazard regression models are presented with hazard ratios (HRs) and 95% CI. To examine whether sex affects the association between cigarette use and hypertension, we further conducted a stratified analysis.

Statistical analyses were performed using R software (version 4.3.2). A p<0.05 on two sides was considered statistically significant.

## RESULTS

### Baseline characteristic of participants

Of 7696 individuals included in this study, the median age was 47 years, and 51.76% of participants were females. Participants with current e-cigarette use, former e-cigarette use, and never e-cigarette use accounted for 5.1%, 14.4%, and 80.6%, respectively. Of 159 e-cigarette-only users, 73 were never combustible cigarette users and 86 were former combustible cigarette users. Current e-cigarette users, with a median age of 32 years, and former e-cigarette users, with a median age of 34 years, were younger than those with never e-cigarette use, with a median age of 50 years. A higher proportion of males was observed in current e-cigarette users compared to those with former and never e-cigarette use. Among never e-cigarette users, married participants accounted for 54.42%, much more than those who were divorced or lived alone. Participants with higher PIR were more likely to never use e-cigarettes. No statistical significance was seen in race/ethnicity, BMI and coronary heart disease among the three e-cigarette statuses. The baseline characteristics are shown in Table 1.

**Table 1:** Baseline characteristics of the study population based on e-cigarette use status, NHANES 2015–2018, United States (N=7696)

*Characteristics*	*Total* *(N=7696)* *n (%)*	*Never e-cigarette* *use* *(N=6196)* *n (%)*	*Former e-cigarette* *use* *(N=1109)* *n (%)*	*Current e-cigarette* *use* *(N=391)* *n (%)*	*p*
**Age** (years), median (IQR)	47.00 (32.00–60.00)	50.00 (36.00–63.00)	34.00 (27.00–48.00)	32.00 (25.00–48.00)	<0.001
**Gender**					<0.001
Female	3962 (51.76)	3295 (53.65)	501 (47.12)	166 (39.43)	
Male	3734 (48.24)	2901 (46.35)	608 (52.88)	225 (60.57)	
**Race**					0.621
Mexican American	1191 (8.51)	1012 (8.69)	135 (8.27)	44 (6.70)	
Non-Hispanic Black	1647 (10.59)	1319 (10.63)	241 (10.39)	87 (10.60)	
Non-Hispanic White	2750 (65.87)	2088 (65.23)	484 (67.71)	178 (69.25)	
Other Hispanic	843 (5.97)	724 (6.20)	90 (5.31)	29 (4.65)	
Other	1265 (9.07)	1053 (9.25)	159 (8.31)	53 (8.79)	
**Marital status**					<0.001
Married	3881 (54.42)	3380 (59.63)	385 (38.47)	116 (29.19)	
Living alone	2960 (35.65)	2125 (30.31)	604 (52.95)	231 (58.89)	
Divorced	855 (9.93)	691 (10.06)	120 (8.58)	44 (11.93)	
**PIR**					<0.001
<1	1519 (12.81)	1170 (11.37)	252 (17.46)	97 (19.15)	
1–2	2102 (19.85)	1653 (18.43)	332 (24.64)	117 (25.52)	
3–4	2093 (28.29)	1680 (28.13)	293 (27.08)	120 (33.74)	
>4	1982 (39.04)	1693 (42.06)	232 (30.83)	57 (21.59)	
**Education level**					<0.001
Lower than high school	676 (3.91)	629 (4.43)	40 (2.16)	7 (1.79)	
High school	824 (7.18)	633 (6.52)	120 (6.95)	71 (16.49)	
Higher than high school	6196 (88.91)	4934 (89.04)	949 (90.89)	313 (81.71)	
**BMI** (kg/m^2^), median (IQR)	28.60 (24.60–33.40)	28.50 (24.70–33.30)	29.10 (24.20–34.10)	27.90 (23.60–33.80)	0.326
**Alcohol use**					<0.001
Former	714 (7.38)	607 (7.78)	77 (5.64)	30 (6.93)	
Heavy	1567 (21.93)	979 (16.16)	425 (41.60)	163 (44.29)	
Mild	2939 (40.65)	2511 (44.64)	324 (26.80)	104 (25.69)	
Moderate	1346 (19.95)	1006 (18.96)	255 (24.04)	85 (21.86)	
Never	1130 (10.10)	1093 (12.46)	28 (1.92)	9 (1.24)	
**DM**					0.003
Yes	1522 (15.11)	1330 (16.29)	145 (10.67)	47 (11.61)	
No	6174 (84.89)	4866 (83.71)	964 (89.33)	344 (88.39)	
**CHD**					0.685
Yes	314 (3.43)	267 (3.48)	33 (2.96)	14 (4.11)	
No	7382 (96.57)	5929 (96.52)	1076 (97.04)	377 (95.89)	
**Combustible cigarette use**					<0.001
Never	4433 (57.37)	4112 (67.69)	248 (22.26)	73 (15.91)	
Former	1796 (25.03)	1447 (24.59)	263 (26.32)	86 (27.31)	
Current	1467 (17.60)	637 (7.72)	598 (51.01)	232 (56.78)	

The p-value was calculated using Pearson’s chi-squared test for continuous variables and the Kruskal–Wallis H test for categorical variables. PIR: poverty-to-income ratio. BMI: body mass index. DM: diabetes mellitus. CHD: coronary heart disease. IQR: interquartile range.

### Association between cigarette use and hypertension

The association between e-cigarette use and hypertension is shown in Table 2. Former e-cigarette use increased by 40.3% the odds of hypertension with never e-cigarette use as reference (Model 3, AOR=1.40; 95% CI: 1.09–1.81). In comparison to never e-cigarette users, current e-cigarette users had 56.3% higher odds of hypertension (Model 3, AOR=1.56; 95% CI: 1.01–2.42). In all three models, the gradient of the association between e-cigarette use and hypertension was statistically significant (all p for trend<0.05).

**Table 2:** Association of e-cigarette use, combustible cigarette use and dual use with hypertension among participants aged ≥20 years, NHANES 2015–2018, United States (N=7696)

*Cigarette use*	*Hypertension*					
	*Model 1*		*Model 2*		*Model 3*	
	*AOR (95% CI)*	*p*	*AOR (95% CI)*	*p*	*AOR (95% CI)*	*p*
**E-cigarette**						
Never ®	1		1		1	
Former	1.65 (1.37–2.00)	<0.001	1.56 (1.27–1.91)	<0.001	1.40 (1.09–1.81)	0.016
Now	1.86 (1.32–2.62)	0.001	1.67 (1.19–2.35)	0.005	1.56 (1.01–2.42)	0.047
p for trend	<0.001		<0.001		0.007	
**Combustible cigarette**						
Never ®	1		1		1	
Former	1.18 (0.99–1.40)	0.057	1.16 (0.97–1.38)	0.096	0.98 (0.80–1.19)	0.776
Now	1.49 (1.25–1.76)	<0.001	1.33 (1.08–1.63)	0.010	1.29 (1.01–1.64)	0.043
p for trend	<0.001		0.005		0.005	
**Dual use**						
Never ®	1		1		1	
Combustible cigarette only	1.42 (1.15–1.76)	0.002	1.26 (0.98–1.63)	0.069	1.25 (0.91–1.73)	0.140
E-cigarette only	1.45 (0.89–2.35)	0.130	1.33 (0.82–2.18)	0.233	1.16 (0.61–2.24)	0.600
Dual use	2.29 (1.44–3.64)	0.001	1.96 (1.19–3.25)	0.012	1.83 (1.03–3.27)	0.042
p for trend	<0.001		0.003		0.030	

AOR: adjusted odds ratio. Model 1: adjusted for age, sex, and race. Model 2: adjusted as for Model 1 plus marital status, education level, and poverty-to-income ratio. Model 3: adjusted as for Model 2 plus body mass index, alcohol use, coronary heart disease, and diabetes mellitus. All the models were weighted logistics regression models that utilized 2-year examination weight (WTMEC2YR). ® Reference categories.

The association between combustible cigarette use and hypertension is shown in Table 2. In Model 1, the prevalence of hypertension was higher in current combustible cigarette users compared to never combustible cigarette users (AOR=1.49; 95% CI: 1.25–1.76). After adjusting for all covariates in the study, the correlation still existed (Model 3, AOR=1.29; 95% CI: 1.01–1.64). The trend of the relationship between combustible cigarette use was significant (all p for trend<0.05). However, the relationship between combustible cigarette use and hypertension was not statistically significant in former and never combustible cigarette users.

The correlation between dual use of e-cigarettes and combustible cigarettes and hypertension is also shown in Table 2. In Model 1, combustible-only cigarette use and dual use increased the odds of hypertension, with never cigarette use as reference. Nevertheless, only dual use of e-cigarettes and combustible cigarettes had a higher probability of having hypertension than never cigarette use, after adjusting for all the covariates in the study (AOR =1.83; 95% CI: 1.03–3.27). Significant trend of association between dual use of e-cigarettes and combustible cigarettes and hypertension was observed (all p for trend<0.05). The study further explored the potential interaction effect of sex on the association between cigarette use and hypertension. No significant interactions were observed between sex and cigarette use (Supplementary file Table 1).

### Associations of use of e-cigarettes, combustible cigarettes, or dual use with all-cause mortality, and cardiovascular mortality in hypertensive individuals

During 9896 person-years of follow-up (median, 38 months), 196 all-cause deaths, including 50 cardiovascular deaths, were documented from NHANES 2015–2018. Proportion hazards assumptions were ascertained using Schoenfeld residual plots and Schoenfeld tests (Supplementary file Figures 1–6). Adjusted Cox regression analysis was performed to assess the relationship of cigarette use and mortality in participants with hypertension, as shown in Table 3. Current e-cigarette use was positively correlated with all-cause death compared to never e-cigarette use (HR=1.30; 95% CI: 1.01–1.66). Additionally, the risk of cardiovascular death increased 29.6% in participants with current e-cigarette use compared to those with never e-cigarette use (HR=1.30; 95% CI: 1.01–1.67). The trends of the association between e-cigarette and all-cause mortality as well as cardiovascular mortality were statistically significant (all p for trend<0.05). While no statistical significance was seen in the relationship of former e-cigarette use and mortality (all-cause death or cardiovascular death), with never e-cigarette use as reference. Meanwhile, current and former combustible cigarette use did not increase hazards of mortality (all-cause death, and cardiovascular death) comparing never combustible cigarette use. Regarding dual use of combustible cigarettes and e-cigarettes, no group had increased risk of mortality. The Kaplan-Meier curves for all-cause mortality and cardiovascular mortality showed no significant difference according to cigarette use status (Supplementary file Figures 7 and 8).

**Table 3:** Association of e-cigarette use, combustible cigarette use and dual use with all-cause mortality and cardiovascular mortality among hypertensive participants aged ≥20 years, NHANES 2015–2018, United States (N=3291)

*Cigarette use*	*All-cause mortality*		*Cardiovascular mortality*	
	*HR (95% CI)*	*p*	*HR (95% CI)*	*p*
**E-cigarette**				
Never ®				
Former	1.04 (0.94–1.15)	0.453	1.04 (0.95–1.15)	0.393
Current	1.30 (1.01–1.66)	0.041	1.30 (1.01–1.67)	0.045
p for trend	0.049		0.044	
**Combustible cigarette**				
Never ®				
Former	1.00 (0.89–1.12)	0.986	1.02 (0.91–1.14)	0.874
Current	0.89 (0.76–1.05)	0.171	0.91 (0.75–1.07)	0.229
p for trend	0.156		0.282	
**Dual use**				
Never ®				
Combustible cigarette only	0.86 (0.72–1.03)	0.095	0.89 (0.75–1.05)	0.139
E-cigarette only	1.27 (0.86–1.87)	0.230	1.28 (0.87–1.89)	0.188
Dual use	1.23 (0.93–1.62)	0.151	1.26 (0.95–1.67)	0.099
p for trend	0.382		0.263	

All the models were adjusted for age, sex, race, marital status, education level, poverty-to-income ratio, body mass index, alcohol use, coronary heart disease, and diabetes mellitus. All the models were weighted Cox proportional hazard regression models that utilized 2-year examination weight (WTMEC2YR).

HR: hazard ratio. ® Reference categories.

## DISCUSSION

In this study utilizing data from NHANES 2015–2018, it was observed that current or former e-cigarette users had higher odds of having hypertension compared to those who had never used e-cigarettes. Furthermore, current use of combustible cigarettes was associated with a higher likelihood of hypertension when compared to individuals who had never used combustible cigarettes. Additionally, individuals with current e-cigarette use had higher hazards of all-cause death or cardiovascular death compared to those with never e-cigarette use.

The acute impact of e-cigarettes on hypertension has been acknowledged through the utilization of different e-cigarette devices. A meta-analysis has indicated a notable increase in systolic and diastolic BP following acute inhalation of e-cigarettes^17^. However, there is limited evidence available regarding the long-term effects of e-cigarette use on hypertension. A previous study examined the relationship between e-cigarette use and metabolic syndrome, defining elevated blood pressure as systolic blood pressure ≥130 mmHg, diastolic blood pressure ≥85 mmHg, or current use of prescribed antihypertensive medications. The results of the population-based study indicated that there was no statistically significant difference in elevated blood pressure between current e-cigarette users and those who had never used e-cigarettes (AOR=0.98; 95% CI: 0.77–1.26), whereas former e-cigarette users had a higher prevalence of elevated BP (AOR=1.20, 95% CI: 1.02–1.42)^18^. Similarly, our study showed that former e-cigarette smokers exhibited higher prevalence of hypertension in comparison to never e-cigarette smokers. However, our study also demonstrated that current e-cigarette users had increased odds of hypertension compared to never e-cigarette users (AOR=1.56; 95% CI: 1.01–2.42). In our study, individuals were seen as having hypertension with systolic BP ≥140 mmHg, diastolic BP ≥90 mmHg, self-reported hypertension, or ever prescribed antihypertensive medications. The discrepancy in the criteria of elevated BP and hypertension may account for the contradicting result.

E-cigarette liquid typically contains propylene glycol, glycerin, distilled water, flavorings (which may not always be approved for food use), and nicotine. Previous research has associated the use of e-cigarettes with endothelial dysfunction, oxidative stress (OS), inflammation, and platelet activation^19^. This may be due to the nicotine contained in the e-liquid and the effects of the aerosol often referred to as vapor, the impacts of which are not yet fully understood. Nicotine has been a known inducer of oxidative stress, causing the imbalance of prooxidants and antioxidants. Playing a role in the generation of reactive oxygen species (ROS), nicotine triggers oxidative stress via multiple pathways, such as the PI3K/Akt-MAPK pathway^20^. Accumulating evidence has indicated that oxidative stress may be involved the onset and progression of hypertension, with ROS acting vitally in the regulation of endothelial function and re-modeling of vascular function^21^. Previous studies illustrated excessive ROS in individuals with hypertension and several hypertensive animal models^22^. Nitric oxide (NO), a key regulator of vascular homeostasis, was reported to be inhibited by NOS in a direct pattern. The inhibition of NO bioavailability could decrease vasodilatory excitability, leading to the onset of hypertension^23^. A randomized controlled study identified the correlation between nicotine and inflammation, such as hs-CRP^24^. Evidence is accumulating that the elevated levels of inflammation are associated with the pathogenesis of hypertension, as well as hypertensive complications^25^. These could potentially be the underlying mechanisms linking current e-cigarette use with hypertension. Given that most individuals start using e-cigarettes after exposure to conventional smoking, and considering a study indicating that approximately 85% of e-cigarette users are dual users, it could potentially explain why former e-cigarette use is associated with increased odds of hypertension^26^.

Prior studies have explored the association between smoking combustible cigarettes and hypertension, focusing on both the immediate and long-term impacts. An early study found that heavy smoking significantly increased BP immediately after smoking one cigarette in individuals without hypertension^27^. The association between long-term use of combustible cigarettes and the development of hypertension has been extensively documented in various studies^28^. In our study, only current combustible users had higher odds of hypertension compared to never combustible cigarette users, in the fully adjusted model. The prevalence of hypertension was similar in former and never cigarette users. These findings might indicate that the cessation of combustible cigarettes provides protective effects on hypertension. In combustible cigarettes, mainstream tobacco smoke comprises >4000 chemical compounds, including a harmful array of free radicals and other ROS and nitrogen species (RNS), which are present in both the gaseous phase and the tar^29^. Cardiovascular damage occurs as a response to key regulators such as OS, inflammation, and alterations in the Nrf2-regulated endogenous antioxidant response system^30^. Concurrently, nicotine in combustible tobacco can also induce OS and vascular endothelial damage, thereby contributing to the development of hypertension^31^.

In our research, individuals with dual use of combustible cigarettes and e-cigarettes were 1.83-fold more likely to have hypertension than never cigarette users after adjusting for potential covariates. A US longitudinal study aimed to examine the prospective association between cigarette use and self-reported incident hypertension. After adjusting for potential confounders, only combustible cigarette use increased risk of self-reported hypertension compared to non-smokers, while dual use or e-cigarette use did not. The category of non-smokers in this study encompassed individuals who were not currently using combustible cigarettes or electronic cigarettes. However, individuals who had previously used electronic or combustible cigarettes were also classified as nonsmokers, which could introduce confounding factors related to smoking cessation. Besides, the diagnosis of hypertension was based on self-reported data rather than precise BP measurements, leading to potential biases. Dual users, who showed higher levels of nicotine dependence and urinary cotinine compared to cigarette-only smokers, also exhibited more psychosocial and behavioral risk factors such as perceived high stress, depressive mood, high daily energy intake, and obesity, compared to never smokers and cigarette-only smokers^32^. This could potentially explain why dual use is associated with a higher odds ratio in comparison to either electronic or combustible cigarette use alone.

Intriguing, the results of our study demonstrated that e-cigarette use increased hazards of all-cause mortality and CV mortality among volunteers with hypertension. Since e-cigarettes were first introduced into the US market in 2007, evidence on the association between e-cigarette use and mortality is limited. Previous researches have demonstrated that the use of e-cigarettes can have significant psychological and physiological impacts on individuals. A study demonstrated that individuals who exclusively utilized e-cigarettes exhibited a higher propensity for suicidal ideation and planning, compared to those who engaged in dual smoking^33^. Furthermore, previous researches suggesting a heightened risk of CV disease associated with e-cigarette use may elucidate the increased hazard ratio for CV mortality^34^. This occurrence could potentially be elucidated by the ‘Splenocardiac Axis’, a mechanism characterized by an inflammatory network that precipitates acute cardiac ischemia. It involves sympathetic nerve activation of hematopoietic tissues like bone marrow and spleen, which release pro-inflammatory monocytes contributing to atherosclerotic plaques and promoting ischemic heart disease^35^. Prospective surveys with long-term follow-up are needed to elucidate the chronic effect of e-cigarettes on mortality.

### Strengths and limitations

The study has several key strengths. First, it was carried out utilizing nationwide representative data with a sophisticated multi-age sampling design, thereby bolstering the stability and reliability of the results. Second, the study employed complex models and weighted complex methodology to examine the relationship between cigarette use and hypertension, as well as all-cause and CV mortality in individuals with hypertension. This methodological approach serves to enhance the precision of the research. Third, in addition to combustible cigarettes and e-cigarettes, we also stratified dual use and assessed the correlation between dual use and hypertension.

There are several limitations in the current study. First, the casual relationship was impossible to infer when assessing the correlation between cigarette use and hypertension due to the cross-sectional nature of the data. Due to limitations of the NHANES database, we were unable to determine the temporal sequence between hypertension onset and the switch to e-cigarette use among individuals who had current e-cigarette use and former combustible cigarette use. Therefore, it remains unclear whether hypertension predated the transition from combustible cigarettes to e-cigarettes or developed as a result of e-cigarette use. Additionally, the increased risk of mortality among e-cigarette users may be due to underlying, pre-existing comorbidities related to prior combustible cigarette use. Second, the reliance on self-reported cigarette use status in interviews introduces potential recall bias. Detailed exposure metrics for vaping and smoking, such as pack-years of smoking, were lacking due to the limitation of the database. Third, given the short duration of e-cigarette utilization, future prospective studies with extended follow-up periods are necessary to investigate the relationship between e-cigarette use and hypertension, as well as mortality in individuals with hypertension. Fourth, although we adjusted for various potential confounders in the models, there is still the possibility of residual confounding due to the limit of NHANES.

## CONCLUSIONS

The findings of this study suggest that current and former e-cigarette use, as well as current combustible cigarette use, are significantly associated with higher odds of hypertension among US adults. Dual users of combustible cigarettes and e-cigarettes have higher odds of hypertension compared to never cigarette users. Additionally, e-cigarette use is correlated with higher hazards of all-cause mortality and CV mortality. Notably, the increased risk of mortality among e-cigarette users may be due to underlying, pre-existing comorbidities related to prior combustible cigarette use. Findings from the study provide evidence of the benefits of e-cigarette use control, especially among individuals with hypertension. Further studies are needed to validate these findings.

## Supplementary Material



## Data Availability

The data supporting this research are available from the following source: https://wwwn.cdc.gov/nchs/nhanes/
