# Supplementary material for: Association of the use of e-cigarettes, combustible cigarettes or dual use with hypertension and mortality in hypertensive individuals: Insights from NHANES 2015–2018
Source: Tob Induc Dis. 2024 Nov 19;22:10.18332/tid/195397. doi: 10.18332/tid/195397 (PMC11574958; doi:10.18332/tid/195397)
Supplement: Supplementary file 1 [file TID-22-178-s1.pdf]

Supplementary Table 1. Association of e-cigarette use, combustible cigarette use and dual use of electronic/combustible cigarette use with hypertension among participants aged ≥ 20 years stratified by sex, NHANES 2015-2018, United States (N = 7696)

Cigarette use	*Male*		*Female*		*p for interaction*
	OR (95% CI)	*p*	*OR (95% CI)*	*p*	
**E-cigarette**					0.075
Never	Ref.		Ref.		
Former	1.59 (1.21, 2.09)		1.44 (0.97, 2.14)	0.070	
Now	1.62 (1.11, 2.36)		1.77 (1.02, 3.08)	0.044	
p for trend	<0.001		0.004		
**Combustible cigarette**					0.676
Never	Ref.		Ref.		
Former	1.07 (0.89, 1.28)	0.443	1.09 (0.82, 1.45)	0.523	
Now	1.56 (1.17, 2.07)	0.004	1.52 (1.03, 2.23)	0.037	
p for trend	0.003		0.039		
**Dual use**					0.413
Never	Ref.		Ref.		
Combustible cigarette only	1.46 (0.98, 2.17)	0.060	1.23 (0.72, 2.10)	0.411	
E-cigarette only	1.30 (0.64, 2.67)	0.433	0.71 (0.28, 1.77)	0.420	
Dual use	1.68 (0.90, 3.14)	0.094	2.17 (1.07, 4.42)	0.036	
p for trend	0.030			0.071	

All the models were adjusted for age, sex, race, marital status, educational levels, poverty-to-income ratio, body mass index, alcohol use, coronary heart disease, and diabetes mellitus. All models were weighted logistic regression models using the 2-year examination weight (WTMEC2YR) as the weight. p for interaction indicates the test for interaction between sex and cigarette use status; no significant interaction was observed. OR: odds ratio. CI: confidence interval. E-cigarette: electronic cigarette. Ref.: reference.

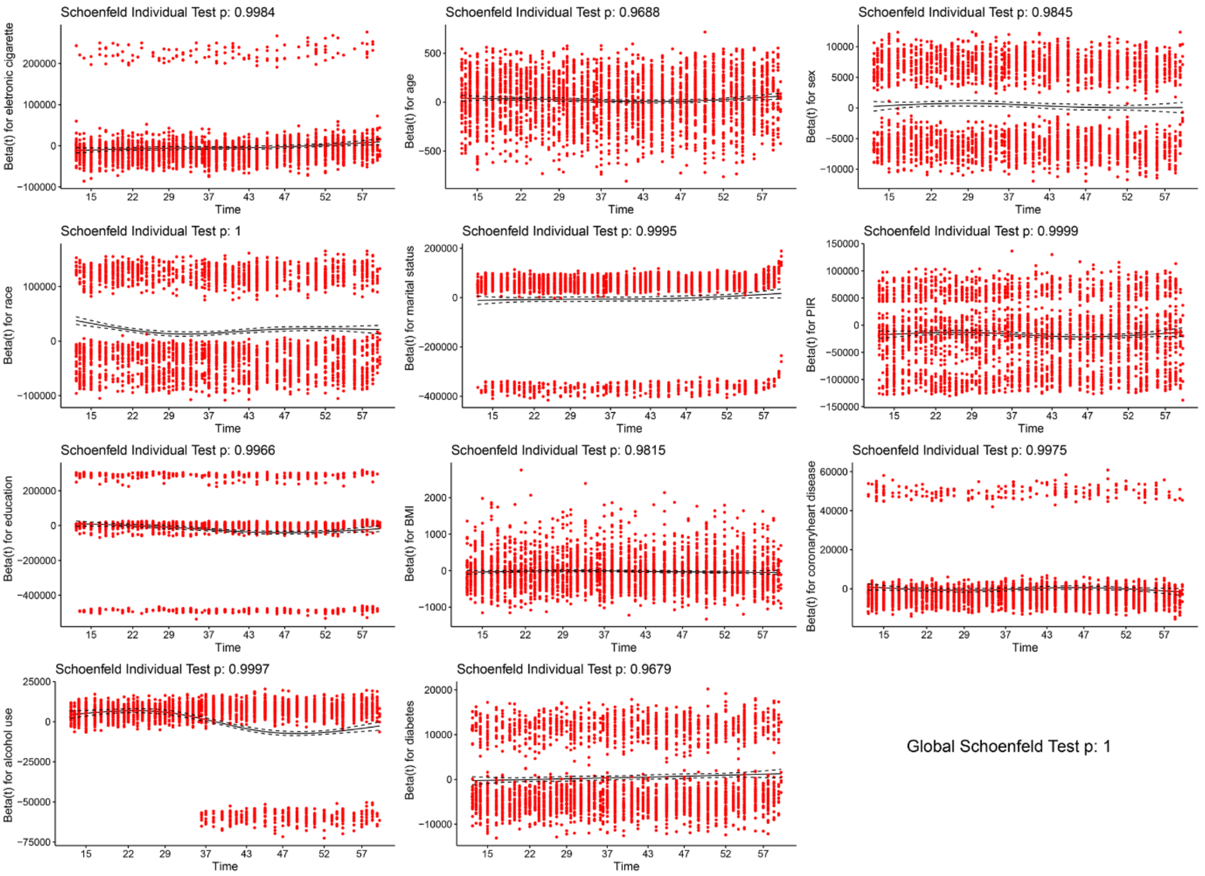

Supplementary Figure 1. Schoenfeld residuals for the proportional hazards assumption in the weighted Cox proportion hazard regression model assessing the association between electronic cigarette use and all-cause mortality among hypertensive participants aged ≥ 20 years, NHANES 2015-2018, United States (N = 3291). The model was adjusted for the following covariates: age, sex, race, marital status, educational levels, PIR, BMI, alcohol use, coronary heart disease, and diabetes. Each plot displays the residuals over time, with the dashed lines representing the 95% confidence intervals. The individual p-values for the Schoenfeld test of each covariate are provided, and the global Schoenfeld test p-value is shown at the bottom, confirming that the model meets the proportional hazards assumption. PIR: poverty-to-income ratio. BMI: body mass index.

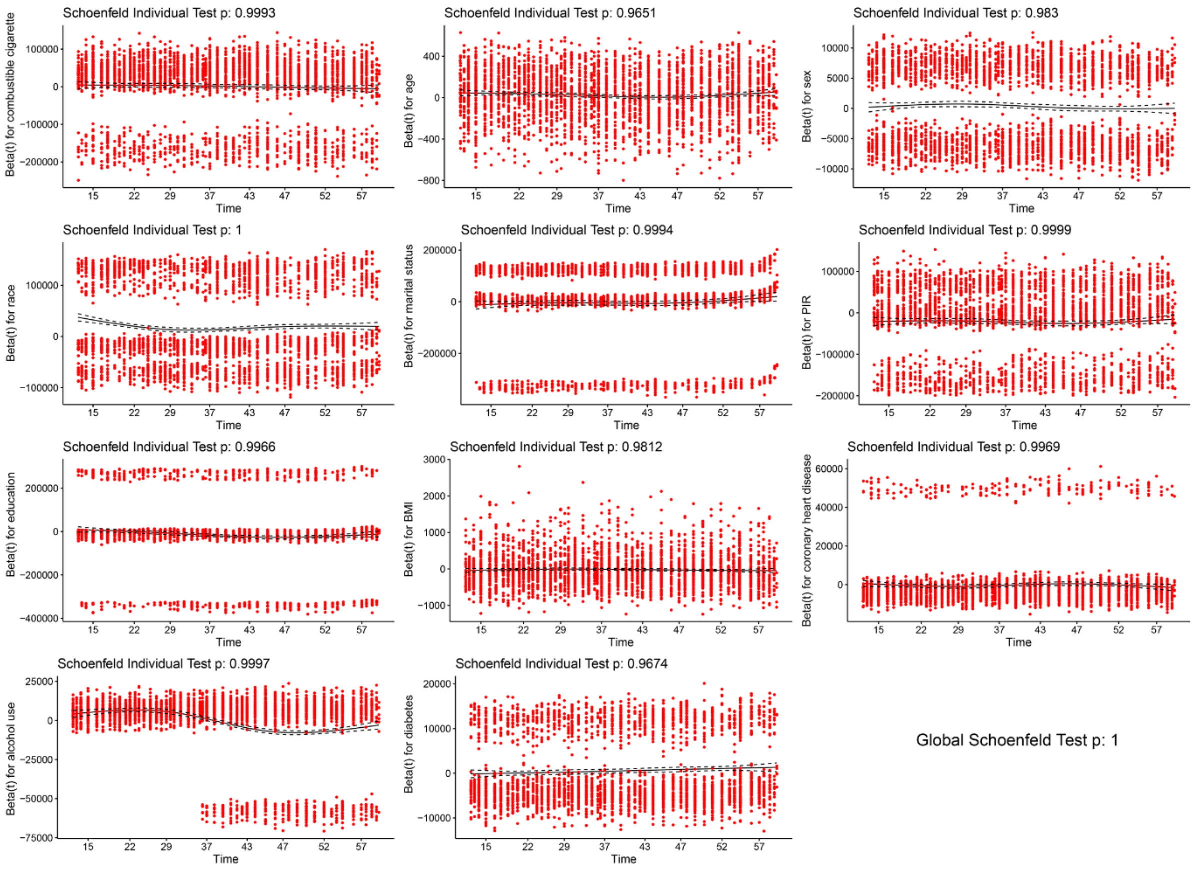

Supplementary Figure 2. Schoenfeld residuals for the proportional hazards assumption in the weighted Cox proportion hazard regression model assessing the association between combustible cigarette use and all-cause mortality among hypertensive participants aged ≥ 20 years, NHANES 2015-2018, United States (N = 3291). The model was adjusted for the following covariates: age, sex, race, marital status, educational levels, PIR, BMI, alcohol use, coronary heart disease, and diabetes. Each plot displays the residuals over time, with the dashed lines representing the 95% confidence intervals. The individual p-values for the Schoenfeld test of each covariate are provided, and the global Schoenfeld test p-value is shown at the bottom, confirming that the model meets the proportional hazards assumption. PIR: poverty-to-income ratio. BMI: body mass index.

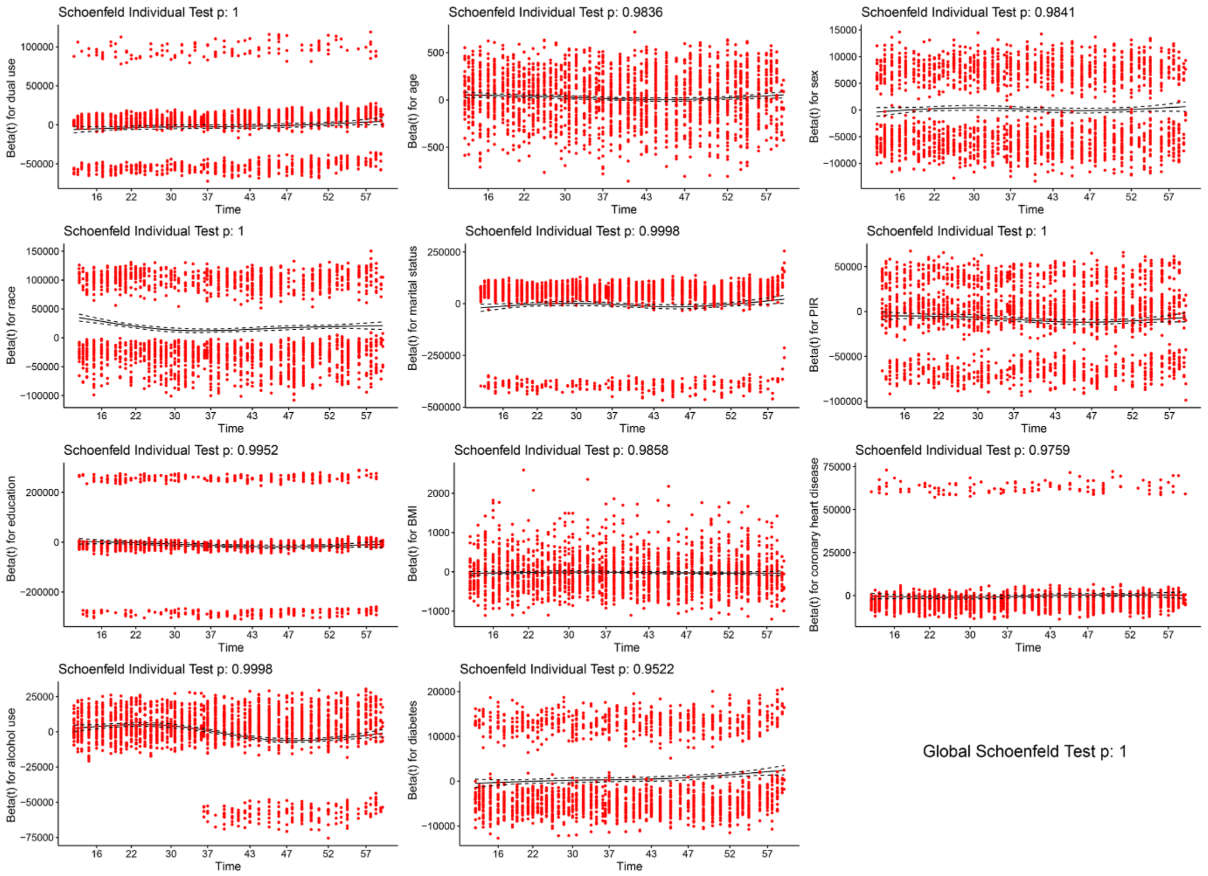

Supplementary Figure 3. Schoenfeld residuals for the proportional hazards assumption in the weighted Cox proportion hazard regression model assessing the association between dual use of electronic cigarette and combustible cigarette and all-cause mortality among hypertensive participants aged ≥ 20 years, NHANES 2015-2018, United States (N = 3291). The model was adjusted for the following covariates: age, sex, race, marital status, educational levels, PIR, BMI, alcohol use, coronary heart disease, and diabetes. Each plot displays the residuals over time, with the dashed lines representing the 95% confidence intervals. The individual p-values for the Schoenfeld test of each covariate are provided, and the global Schoenfeld test p-value is shown at the bottom, confirming that the model meets the proportional hazards assumption. PIR: poverty-to-income ratio. BMI: body mass index.

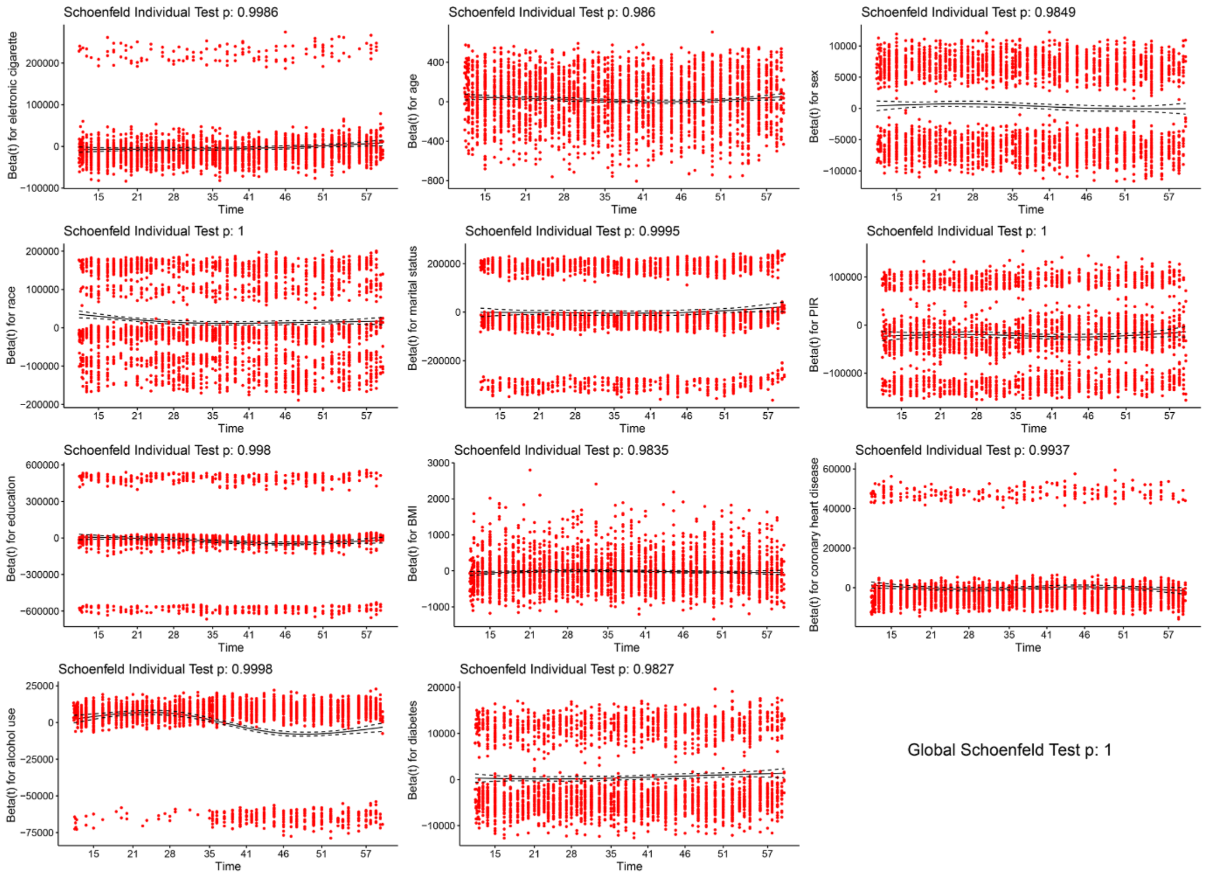

Supplementary Figure 4. Schoenfeld residuals for the proportional hazards assumption in the weighted Cox proportion hazard regression model assessing the association between electronic cigarette use and cardiovascular mortality among hypertensive participants aged ≥ 20 years, NHANES 2015-2018, United States (N = 3291). The model was adjusted for the following covariates: age, sex, race, marital status, educational levels, PIR, BMI, alcohol use, coronary heart disease, and diabetes. Each plot displays the residuals over time, with the dashed lines representing the 95% confidence intervals. The individual p-values for the Schoenfeld test of each covariate are provided, and the global Schoenfeld test p-value is shown at the bottom, confirming that the model meets the proportional hazards assumption. PIR: poverty-to-income ratio. BMI: body mass index.

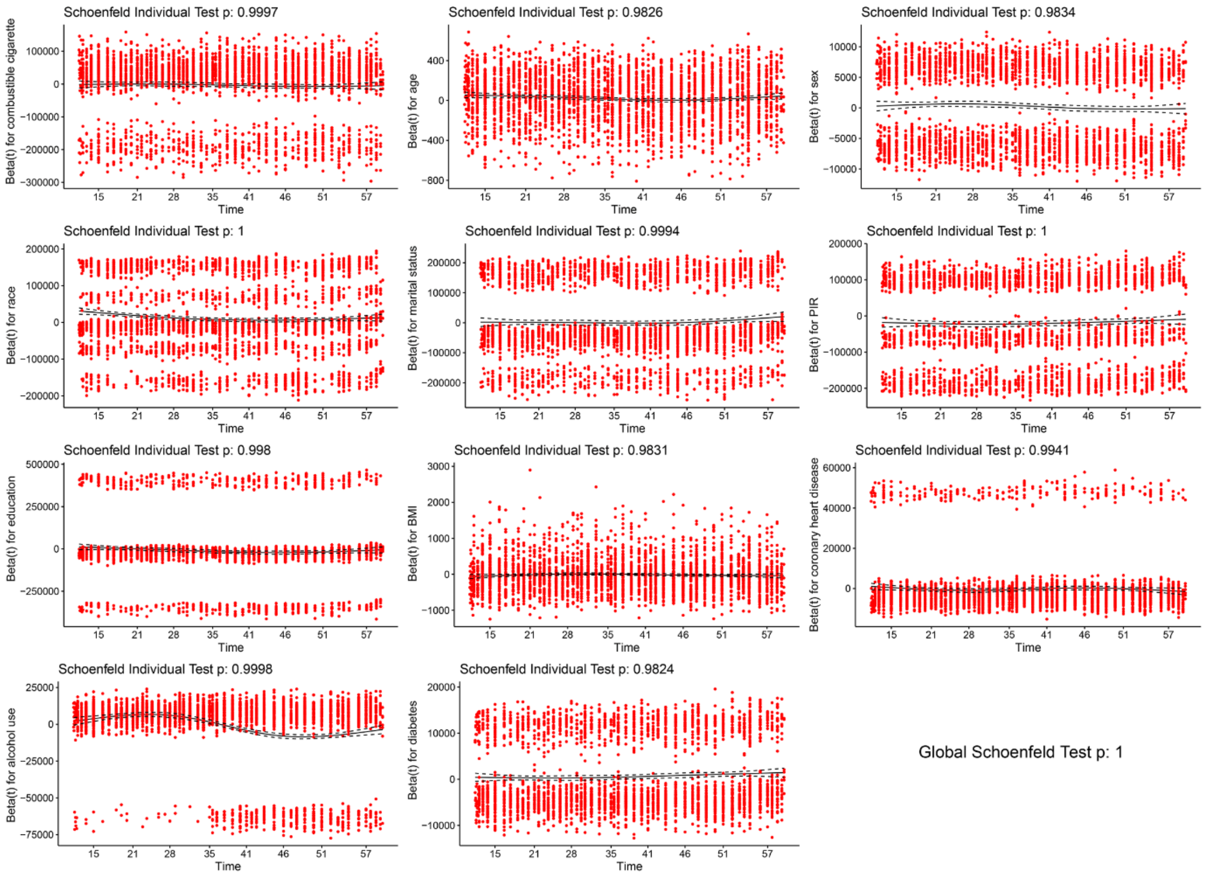

Supplementary Figure 5. Schoenfeld residuals for the proportional hazards assumption in the weighted Cox proportion hazard regression model assessing the association between combustible cigarette use and cardiovascular mortality among hypertensive participants aged ≥ 20 years, NHANES 2015-2018, United States (N = 3291). The model was adjusted for the following covariates: age, sex, race, marital status, educational levels, PIR, BMI, alcohol use, coronary heart disease, and diabetes. Each plot displays the residuals over time, with the dashed lines representing the 95% confidence intervals. The individual p-values for the Schoenfeld test of each covariate are provided, and the global Schoenfeld test p-value is shown at the bottom, confirming that the model meets the proportional hazards assumption. PIR: poverty-to-income ratio. BMI: body mass index.

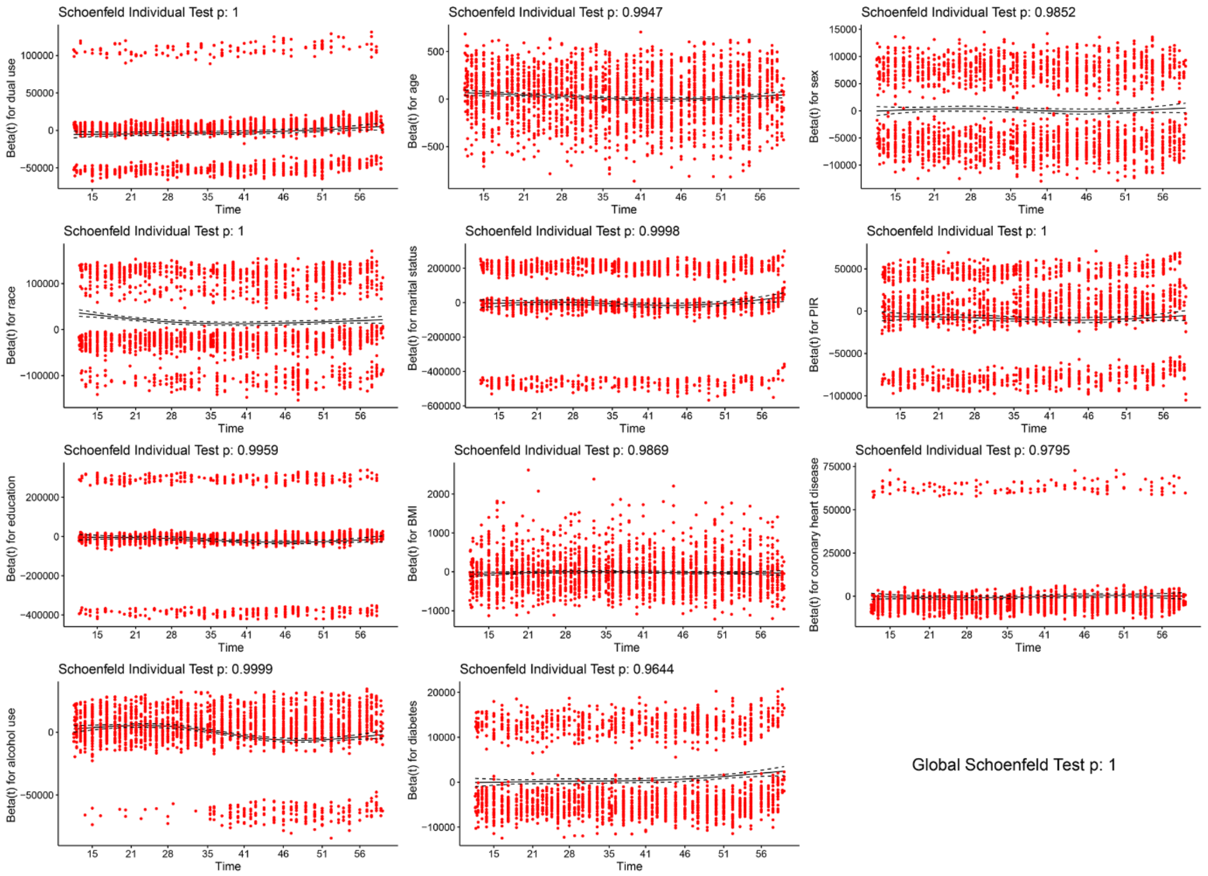

Supplementary Figure 6. Schoenfeld residuals for the proportional hazards assumption in the weighted Cox proportion hazard regression model assessing the association between dual use of electronic cigarette and combustible cigarette and cardiovascular mortality among hypertensive participants aged ≥ 20 years, NHANES 2015-2018, United States (N = 3291). The model was adjusted for the following covariates: age, sex, race, marital status, educational levels, PIR, BMI, alcohol use, coronary heart disease, and diabetes. Each plot displays the residuals over time, with the dashed lines representing the 95% confidence intervals. The individual p-values for the Schoenfeld test of each covariate are provided, and the global Schoenfeld test p-value is shown at the bottom, confirming that the model meets the proportional hazards assumption. PIR: poverty-to-income ratio. BMI: body mass index.

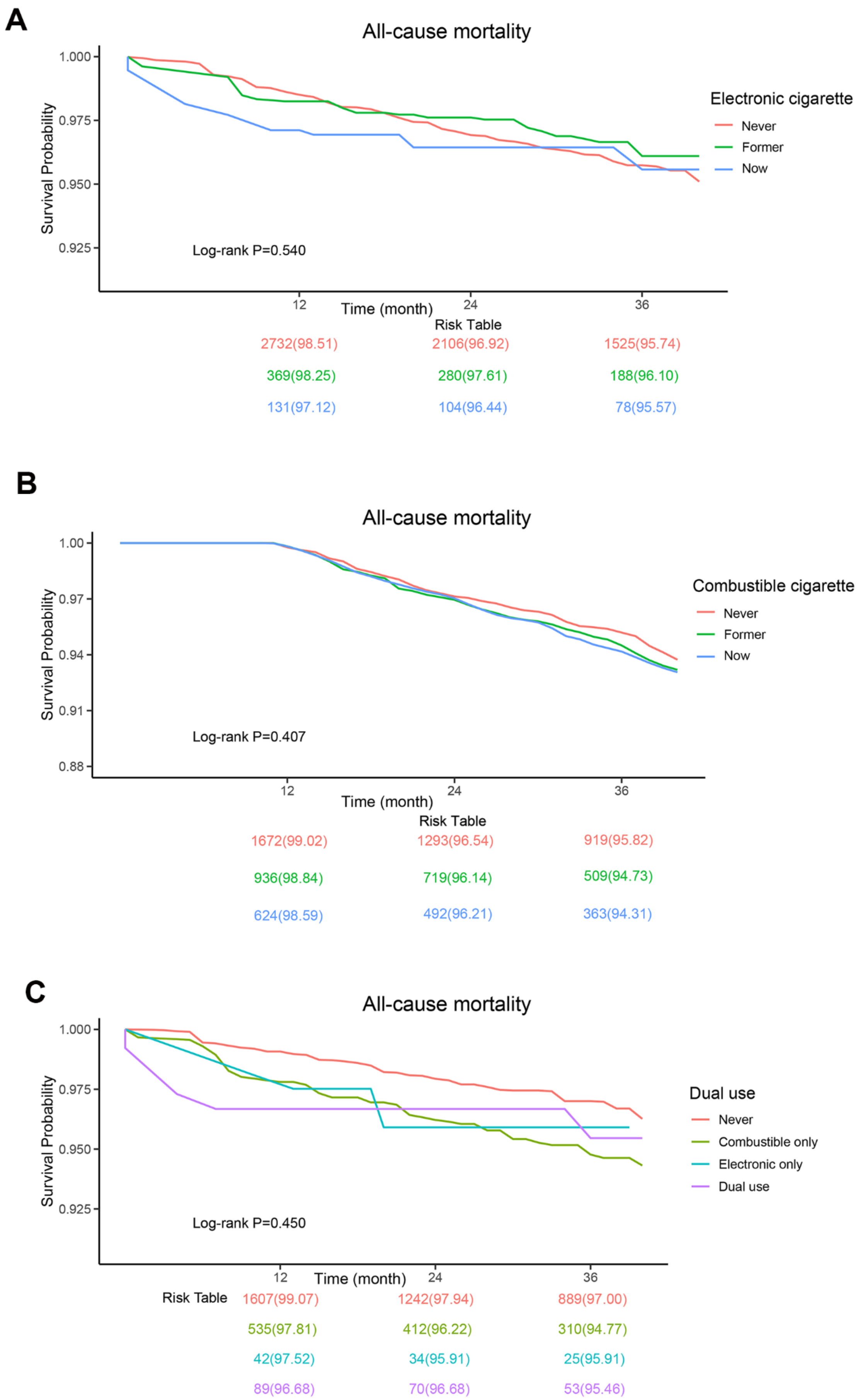
A
All-cause mortality
Survival Probability
1.000
0.975
0.950
0.925
Log-rank P=0.540
12
24
36
Time (month)
Electronic cigarette
Never
Former
Now
Risk Table
2732(98.51) 2106(96.92) 1525(95.74)
369(98.25) 280(97.61) 188(96.10)
131(97.12) 104(96.44) 78(95.57)
B
All-cause mortality
Survival Probability
1.00
0.97
0.94
0.91
0.88
Log-rank P=0.407
12
24
36
Time (month)
Combustible cigarette
Never
Former
Now
Risk Table
1672(99.02) 1293(96.54) 919(95.82)
936(98.84) 719(96.14) 509(94.73)
624(98.59) 492(96.21) 363(94.31)
C
All-cause mortality
Survival Probability
1.000
0.975
0.950
0.925
Log-rank P=0.450
12
24
36
Time (month)
Dual use
Never
Combustible only
Electronic only
Dual use
Risk Table
1607(99.07) 1242(97.94) 889(97.00)
535(97.81) 412(96.22) 310(94.77)
42(97.52) 34(95.91) 25(95.91)
89(96.68) 70(96.68) 53(95.46)

Supplementary Figure 7. Kaplan-Meier curve for all-cause mortality among hypertensive participants aged ≥ 20 years, NHANES 2015-2018, United States (N = 3291). (**A**) Electronic cigarette use. **(B)** Combustible cigarette use. (**C)** Dual use of electronic and combustible cigarettes. Log-rank p-values are provided for each comparison, with risk tables shown below the curves indicating the number (percentage) of participants at risk over time in each group.

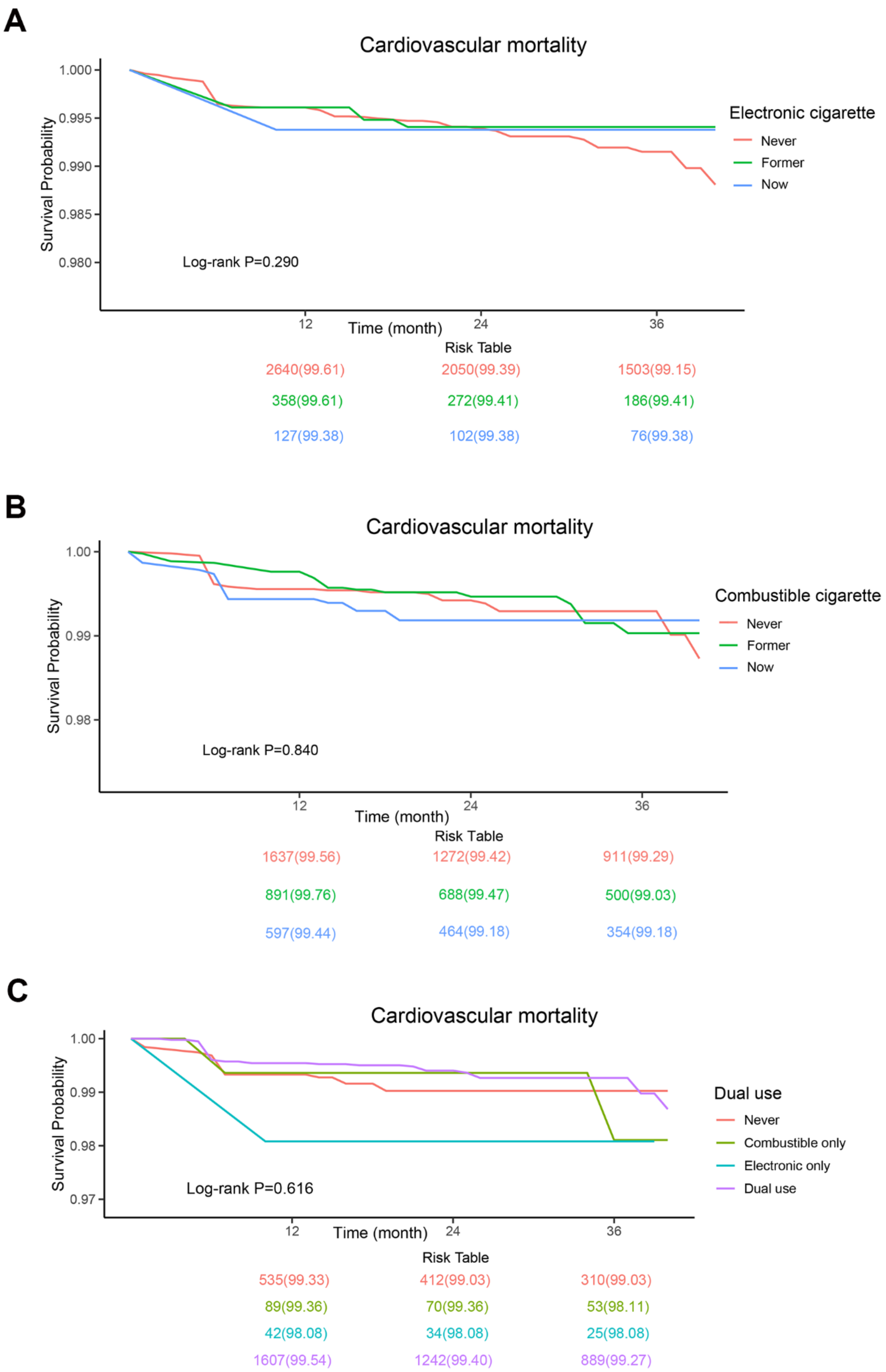
A
Cardiovascular mortality
Survival Probability
1.000
0.995
0.990
0.985
0.980
Log-rank P=0.290
12
Time (month)
24
36
Electronic cigarette
Never
Former
Now
Risk Table
2640(99.61)
2050(99.39)
1503(99.15)
358(99.61)
272(99.41)
186(99.41)
127(99.38)
102(99.38)
76(99.38)
B
Cardiovascular mortality
Survival Probability
1.00
0.99
0.98
Log-rank P=0.840
12
Time (month)
24
36
Combustible cigarette
Never
Former
Now
Risk Table
1637(99.56)
1272(99.42)
911(99.29)
891(99.76)
688(99.47)
500(99.03)
597(99.44)
464(99.18)
354(99.18)
C
Cardiovascular mortality
Survival Probability
1.00
0.99
0.98
0.97
Log-rank P=0.616
12
Time (month)
24
36
Dual use
Never
Combustible only
Electronic only
Dual use
Risk Table
535(99.33)
412(99.03)
310(99.03)
89(99.36)
70(99.36)
53(98.11)
42(98.08)
34(98.08)
25(98.08)
1607(99.54)
1242(99.40)
889(99.27)

Supplementary Figure 8. Kaplan-Meier curve for all-cause mortality among hypertensive participants aged ≥ 20 years, NHANES 2015-2018, United States (N = 3291). (**A**) Electronic cigarette use. **(B)** Combustible cigarette use. (**C)** Dual use of electronic and combustible cigarettes. Log-rank p-values are provided for each comparison, with risk tables shown below the curves indicating the number (percentage) of participants at risk over time in each group.

© 2024 Lu Y. et al.
